# Simo decoction curing spleen deficiency constipation was associated with brain-bacteria-gut axis by intestinal mucosal microbiota

**DOI:** 10.3389/fmicb.2023.1090302

**Published:** 2023-02-09

**Authors:** Xin Yi, Kang Zhou, Na Deng, Ying Cai, Xinxin Peng, Zhoujin Tan

**Affiliations:** ^1^The Domestic First-class Discipline Construction Project of Chinese Medicine, Hunan University of Chinese Medicine, Changsha, China; ^2^The First Affiliated Hospital of Hunan University of Chinese Medicine, Changsha, China

**Keywords:** spleen deficiency constipation, intestinal mucosal microbiota, simo decoction, *Folium sennae* decoction, diet and water intake, oxidative stress, brain-bacteria-gut axis

## Abstract

**Background:**

Simo decoction (SMD) is a traditional prescription for treating gastrointestinal diseases. More and more evidences prove that SMD can treat constipation by regulating intestinal microbiota and related oxidative stress indicators, but the specific mechanism is still unclear.

**Methods:**

A network pharmacological analysis was used to predict the medicinal substances and potential targets of SMD to alleviate constipation. Then, 15 male mice were randomly divided into normal group (MN group), natural recovery group (MR group), and SMD treatment group (MT group). Constipation model mice were constructed by gavage of *Folium sennae* decoction and control of diet and drinking water, and SMD was used for intervention after successful modeling. The levels of 5-hydroxytryptamine (5-HT), vasoactive intestinal peptide (VIP), superoxide dismutase (SOD), malondialdehyde (MDA), and fecal microbial activities were measured, and the intestinal mucosal microbiota was sequenced.

**Result:**

Network pharmacology analysis showed that a total of 24 potential active components were obtained from SMD, and 226 target proteins were obtained after conversion. Meanwhile, we obtained 1,273 and 424 disease-related targets in the GeneCards database and the DisGeNET database, respectively. After combination and deduplication, the disease targets shared 101 targets with the potential active components of SMD. When the mice were intervened with SMD, the 5-HT, VIP, MDA, SOD content, and microbial activity in MT group were close to MN group, and Chao 1 and ACE in MT group were significantly higher than that in MR group. In the Linear discriminant analysis Effect Size (LEfSe) analysis, the abundance of beneficial bacteria such as *Bacteroides*, *Faecalibacterium*, *Alistipes*, *Subdoligranulum*, *Lactiplantibacillus*, and *Phascolarctobacterium* in MT group increased. At the same time, there were some associations between microbiota and brain-gut peptides and oxidative stress indicators.

**Conclusion:**

SMD can promote intestinal health and relieve constipation through brain-bacteria-gut axis associating with intestinal mucosal microbiota and alleviate oxidative stress.

## 1. Introduction

Constipation is a common digestive system disorder that affects many patients worldwide, poses a huge economic burden, and leads to significant healthcare utilization (Sharma and Rao, 2017; Milosavljevic et al., 2022). Patients with constipation usually present with reduced frequency of bowel movements, hard or lumpy stools, incomplete bowel movements, abdominal distention, and pain (Zhao et al., 2022). Slow-transmission constipation is the main type of constipation, which is characterized by a decreased colonic transmission rate (Tian et al., 2021). While slow-transmission constipation belongs to spleen deficiency constipation in Traditional Chinese Medicine (TCM), Spleen deficiency syndrome is one of the factors for the formation of constipation, of which spleen *Qi* deficiency is more common, it will affect the digestion and absorption of food, as well as the formation and spread of feces (Yu et al., 2022). In TCM, it is believed that spleen and stomach are the root of after birth and the source of *Qi* and blood, which are closely related to digestion, absorption and immune defense of the body. When the spleen and stomach are weak, digestion and absorption will decrease, accompanied by blockage of *Qi* movement, which will further affect the promotion of the large intestine, weak and slow the intestinal transmission, and finally form spleen deficiency constipation. Therefore, TCM often associates spleen deficiency constipation with slow transit constipation (Yu et al., 2022).

Studies have found that constipation is closely related to the mental factors of patients, and people who have constipation for a long time have higher anxiety, causing gastrointestinal disorders, and are more likely to aggravate constipation. This relationship between mental factors and intestinal interaction is the brain-gut axis (Chen et al., 2022). Brain-gut peptides are a kind of small molecule polypeptide substances secreted by endocrine cells of brain-gut axis, which are doubly distributed in the gastrointestinal tract and nervous system, and actively active in various pathways of brain-gut axis to achieve brain-gut interaction (Chen and Du, 2019). Among them, 5-HT and VIP, as important brain-gut peptides mediating the brain-gut axis pathway, are not only closely related to the occurrence of constipation, but also to intestinal microbiota. Short-chain fatty acids produced by intestinal bacteria directly stimulate tryptophan hydroxylase 1, leading to the synthesis and secretion of 5-HT in intestinal pheochromocytoma (Martin et al., 2018; Margolis et al., 2021). And VIP will participate in the process of intestinal microbiota imbalance (Xu et al., 2017). In recent years, more and more researchers have focused on the relationship between intestinal microbiota and constipation, and have confirmed the involvement of intestinal microbiota in the pathophysiological processes of gastrointestinal diseases. Long-term accumulation of feces in the intestine during constipation will change the structure of the intestinal microbiota (Zhang et al., 2021). Constipation also stimulates the intestinal mucosal barrier and destroys the immune system. Constipation will reduce immune function and lead to intestinal dysfunction (Zhang et al., 2021). In addition, the imbalance of intestinal microbiota will also affect the intestinal motility of the host, increase the harmful bacteria and endotoxin in the intestinal tract, and thus exacerbate constipation (Fu et al., 2021). However, the complex interaction between intestinal flora and brain-gut axis is called the brain-bacteria-gut axis (Shandilya et al., 2021). In addition, oxidative stress may also be related to the occurrence and development of constipation. SOD and MDA are commonly used indicators to measure the extent of oxidative stress damage. Studies have found that if the body is damaged by oxidative stress, it will produce a strong cytotoxic effect, damaging the intestinal wall mucosal cells to cause intestinal mucosal dysfunction, and ultimately aggravating constipation (Cai et al., 2021).

SMD was used to treat gastrointestinal diseases for hundreds of years (Cai et al., 2011; Luo et al., 2021). The modern SMD oral liquid comes from the Dou zhen jin jing lu in the ancient prescription of Ming Dynasty. And was composed of four herbs, *Citrus aurantium* L. (CL), *Aucklandia lappa* Decne. (AD), *Areca catechu* L. (AL), and *Lindera aggregata* (Sims) Kos-term. (LK) (Deng et al., 2022). SMD is an agent for regulating *Qi*, which can make *Qi* movement unobstructed, gradually restore the propulsion function of large intestine, ameliorate *Qi* deficiency, and relieve constipation (Wang et al., 2021). Among them, AD is capable of moving *Qi* and invigorating spleen, and spleen *Qi* will be enriched after *Qi* movement is smooth. CL can help AD to smooth the operation of *Qi*. Later, AL and LK are used to regulate *Qi* and promote intestinal peristalsis (Huang et al., 2022). The combination of these drugs can enhance the immunity, invigorate the spleen and replenish *Qi*. Furthermore, it is found that SMD can restore and improve gastrointestinal motility by promoting gastric emptying and regulating hormones related to gastrointestinal motility (Li et al., 2020; Yan et al., 2022). SMD could also treat constipation by improving the transcription level of genetic information of intestinal microbiota, increasing tyrosine-protein kinase growth factor receptors, and protecting the intestinal mucosal barrier (Dai et al., 2013; He et al., 2017; Fan et al., 2018; Hui et al., 2018; Xiao and Liu, 2018).

Therefore, we predicted the effective components and potential targets of SMD in treating constipation through network pharmacology, and then investigated gastrointestinal hormones, oxidative stress indexes and intestinal mucosal microbiota in mice with spleen deficiency constipation under the intervention of SMD. It will provide a new idea and research basis for constipation treatment and clinical use of SMD.

## 2. Materials and methods

### 2.1. Materials

#### 2.1.1. Animals

To exclude the effect of gender on intestinal microbiota (Wu et al., 2022), male SPF-grade Kunming mice were selected for this study. Mice were purchased from Hunan Slaccas Jingda Laboratory Animal Company (Hunan, China) with license number SCXK (Xiang)-2019–0004.

#### 2.1.2. Feed

General feed: Mice feed was provided by the Experimental Animal Center of Hunan University of Chinese Medicine and produced by Jiangsu Medison Biomedical Co., LTD. The main indicators of nutrient composition include moisture, crude protein, crude fiber, crude fat, crude ash, calcium, total phosphorus, lysine, methionine, and cystine.

Low-fiber feed: Rice, produced by COFCO Rice Co., LTD. The main indicators of nutrient content include energy, protein, carbohydrate, and sodium.

#### 2.1.3. Drug

*Folium sennae* (140 g) were purchased from the First Affiliated Hospital of Hunan University of Chinese Medicine, soaked in 10 times of boiling water for 10 min, filtered. The filtrate was concentrated into 100% (1 g/ml raw drug) aqueous decoction in a rotary evaporator at 75°C and stored at 4°C for backup. SMD oral liquid was manufactured by Hunan Hansen Pharmaceutical Co., LTD (State drug approval document No. Z20025044, Batch production No. 2112148), the quality standard follows good manufacturing practice.

### 2.2. Methods

#### 2.2.1. Network pharmacological analysis of SMD—constipation

When active ingredients and potential targets of SMD were screened, active ingredients and potential targets were collected including active ingredients of CL, AD, AL, and LK in the compound preparation of traditional Chinese medicine (Li et al., 2021a).

When potential targets for constipation were screened, we searched the DisGeNET database^1^ and the GeneCards database^2^ by using the keyword “constipation” to retrieve relevant targets. After the targets with a systematic score < 0.01 from the DisGeNET database and those with a systematic score < 35 from the GeneCards database were eliminated, the remaining targets were merged, de-duplicated and formatted to obtain potential constipation targets. Venny 2.1.0 online software mapping tool platform was used to identify the intersection target between constipation and SMD (Zhou et al., 2022).

#### 2.2.2. Animal grouping

After 3 days of adaptive housing in a suitable environment (temperature 23 ~ 25°C, relative humidity 50 ~ 70%, clean and quiet), the mice were randomly divided into normal group (MN group), natural recovery group (MR group), and SMD treatment group (MT group), 5 mice per group. All experiments and procedures involving animals were performed following the protocol approved by the Institutional Animal Care and Use Committee of the Hunan University of Chinese Medicine.

#### 2.2.3. Molding and drug administration

Spleen deficiency caused by diarrhea of bitter cold is currently the relatively stable modeling method for spleen deficiency model. *Folium sennae* belongs to bitter cold herbs. As the bitter cold herbs cause diarrhea, they will cause the loss of a large number of body fluids, leading to the exhaustion of *Qi* along with the body fluid. After long-term administration, it is easy to cause spleen and stomach dysfunction, resulting in spleen deficiency (Li et al., 2022a). At the same time, according to the theory of TCM, excessive hunger and thirst can also weaken the spleen and stomach (Jiang et al., 2018). In summary, the spleen deficiency factor was continued to be applied by limiting drinking water and low-fiber feed, and causes constipation.

From 1 ~ 7 days, MR and MT groups were gavaged with *Folium sennae* decoction 0.8 ml/d, twice a day, 0.4 ml each time, causing spleen deficiency, while MN group was gavaged with equal doses of sterile water. From 8 ~ 15 days, stopping the gavage of *Folium sennae* decoction, MN group was fed with normal diet and water, and MR and MT groups were fed with a low-fiber diet of raw rice 4 ~ 8 g, and water was freely drunk once for 30 min each time. The constipation model was created by using water restriction and diet control methods based on the spleen deficiency model, which caused spleen deficiency constipation for a total of 15 days (Zou et al., 2009). When the mice showed lusterless dorsal fur, tiredness, lethargy, weakness of limbs, arching back, thinness, and dryness, it meant the spleen deficiency model mice were successful. When the number of feces decreased, the pellets became smaller and harder, and the above symptoms of spleen deficiency lasted, it means the spleen deficiency constipation model mice were successful (Zou et al., 2009). After modeling, from 16 ~ 22 days, MT group was gavaged with 0.05 g/kg·d SMD oral liquid, and MN and MR groups were gavaged with equal doses of sterile water for a total of 7 days (Li et al., 2015).

#### 2.2.4. General characteristics

The general conditions of each group of mice before and after modeling were observed separately, including body weight, food intake, activity, mental status, and fecal status. Meanwhile, fresh feces from each group before modeling, at the end of *Folium sennae* gavage (7 days), modeling (15 days), and SMD intervention (22 days) were collected to calculate the fecal water content. The fresh feces were dried to constant weight and weighed to record the dry weight to calculate the fecal water content. Fecal water content (%) = (wet weight - dry weight)/wet weight ×100% (Cao et al., 2017).

#### 2.2.5. Fecal microbial activity

Fresh feces were collected in 1.5 ml sterile ep tubes and stored at −20°C from each mouse at 14:00. Pipette 2.5 ml Fluorescein diacetate (FDA) storage solution in prepared phosphate buffer solution to make A solution. Add 50 μl of sample solution to the tube containing 2 ml A solution, shake at 24°C for 90 min, add 2 ml of acetone to terminate the reaction, and measure each sample three times in parallel. In addition, 100 μl of the sample solution and 2 ml of acetone were added to the tube containing 2 ml A solution in turn, and the reaction was carried out at 24°C for 90 min on a shaking bed as a blank control, and the A_490_ nm value was measured (Wu et al., 2021).

#### 2.2.6. Determination of serum 5-HT and VIP levels

Blood was collected from the eyes of mice under sterile conditions. The blood samples were allowed to stand at room temperature for 2 h. The supernatant was centrifuged at low temperature and high speed (4°C, 3000 r/min) for 10 min. The supernatant was used for the determination of 5-HT and VIP. The enzyme-linked immunosorbent assay (ELISA) was used to determine the levels of 5-HT and VIP in serum samples, and the specific operation was performed according to the kit instructions. The VIP and 5-HT kits were provided by Quanzhou kenuodi Biotechnology Co., Ltd.

#### 2.2.7. Determination of liver homogenate MDA and SOD levels

Mice were executed using cervical dislocation on a sterile operating platform. The livers were removed, two 0.1 g liver tissues were weighed. 1 ml MDA and SOD were added to the ice bath homogenate, and centrifuged at 8000 r/min for 10 min at 4°C. The liver homogenate MDA and SOD levels were determined by enzyme-linked immunosorbent assay, according to the kit instructions. The MDA and SOD kits were provided by Beijing Solarbio Science ﹠Technology Co., LTD.

#### 2.2.8. Spleen and thymus indices

The intact spleen and thymus were removed and the attached surface fascia and adipose tissue were removed. The blood on the surface of the viscera was blotted with filter paper and weighed to calculate the spleen and thymus indices: Visceral index = visceral weight (mg)/body weight (g) (Li et al., 2022).

#### 2.2.9. Intestinal mucosa samples from mice

After mice were executed by the cervical dislocation method on a sterile operating table, the jejunum to ileum segment was taken as the test specimen. After extrusion of the contents, the intestinal tissue was slit and rinsed clean with normal saline, the saline on the intestinal wall was blotted with filter paper, and the intestinal mucosa was scraped off with a coverslip and placed in centrifuge tubes and stored at −80°C for backup (Zhang et al., 2020).

#### 2.2.10. PCR amplification and 16S rRNA Illumina NovaSeq sequencing

DNA was extracted from 15 samples using the Tguide S96 kit, and DNA was assayed for concentration using an enzyme marker (manufacturer: Gene Co., Ltd. model: synergy HTX). The extracted DNA was used as a template to amplify the V3 + V4 variable region of bacterial 16S rDNA with the forward primer 338F (5’-ACTCCTACGGGAGGCAGCA-3′) and the reverse primer 806R (5′- GGACTACHVGGGTWTCTAAT-3′) under the following amplification conditions: 5 μl KOD FX Neo buffer, 0.3 μl (10 μ M) of forward and reverse primers, 2 μl (2 mM) of dNTPs, 0.2 μl of KOD FX Neo, and 5–50 ng of DNA template, ddH2O supplemented to 10 μl Reaction conditions. Pre-denaturation at 95°C for 3 min, followed by 25 cycles at 95°C for 30 s, annealing at 50°C for 30 s, extension at 72°C for 40 s, and then extension at 72°C for 7 min. After amplification PCR products were examined for integrity by electrophoresis using agarose at a concentration of 1.8%, and DNA concentration and purity were determined using a NanoDrop 2000 (Thermo Scientific, United States). The PCR products were then sequenced using Illumina’s NovaSeq 6,000 platform, PE250 sequencing strategy. Sample DNA extraction, amplification, and library sequencing were performed by Beijing Biomarker Technologies Co, LTD.

#### 2.2.11. Bioinformatics

Sequences were clustered at the 97% similarity level using USEARCH 10.0 (Edgar, 2013), and Operational taxonomic units (OUT) were filtered by default using 0.005% of the number of all sequences sequenced as a threshold (Bokulich et al., 2013). Alpha indices such as ACE, Chao1, Shannon, and Simpson were analyzed by using QIIME2^3^ (Li et al., 2021b). Principal component analysis (PCA) of samples was plotted based on the R language platform (Li et al., 2021c). LefSe analysis of species differ significantly between groups, by using linear discriminant analysis (LDA) to estimate the effect of the abundance of each component (species) on the effect of the difference (Segata et al., 2011). Based on the abundance and variation of each species in each sample, Spearman’s rank correlation analysis was performed and correlation networks were constructed by screening data with correlations greater than 0.1 and *p*-values less than 0.05. Kyoto Encyclopedia of Genes and Genomes (KEGG) functional analysis by comparing 16S rRNA gene sequence data from the Silva database. The known gut microbiota composition gene functional mapping database was “mapped” to predict the metabolic function of the bacterial microbiota (Li et al., 2022b).

#### 2.2.12. Statistical analysis

The results were expressed as the mean ± standard deviation. GraphPad Prism 9 was used for bar graphs and SPSS 25.0 software was used for statistical analysis. The data differences between groups were compared by independent sample *t*-test. *p* < 0.05 indicates a significant difference, and *p* < 0.01 indicates an extremely significant difference. The values of *p* are as follows: *p < 0.05, **p < 0.01, ***p < 0.001 (Zhang et al., 2021).

## 3. Results and analysis

### 3.1. Network pharmacological analysis of SMD—constipation

#### 3.1.1. Collection of effective ingredients and corresponding targets of SMD

SMD contains the four herbs CL, AD, AL, and LK, and we collected the active ingredients in CL, AD, AL, and LK in the TCMSP^4^. According to bioavailability (OB) ≥ 30% and drug-likeness (DL) ≥ 0.18 as the screening conditions, the effective ingredients of SMD were filtered. The effective ingredients obtained were 8 in LK, 7 in AL, 6 in CL, and 5 in AD, yielding a total of 26 candidate active compound components, of which both LK and AD possessed sitosterol and both LK and CL possessed beta-sitosterol. After de-duplication, 24 targets of effective ingredients in SMD were converted by the UniProt database and protein standardized naming was carried out. After de-duplication, 226 target proteins were finally obtained (Zhou et al., 2022).

#### 3.1.2. Collection of targets for the treatment of constipation with SMD

**Figure 1:**
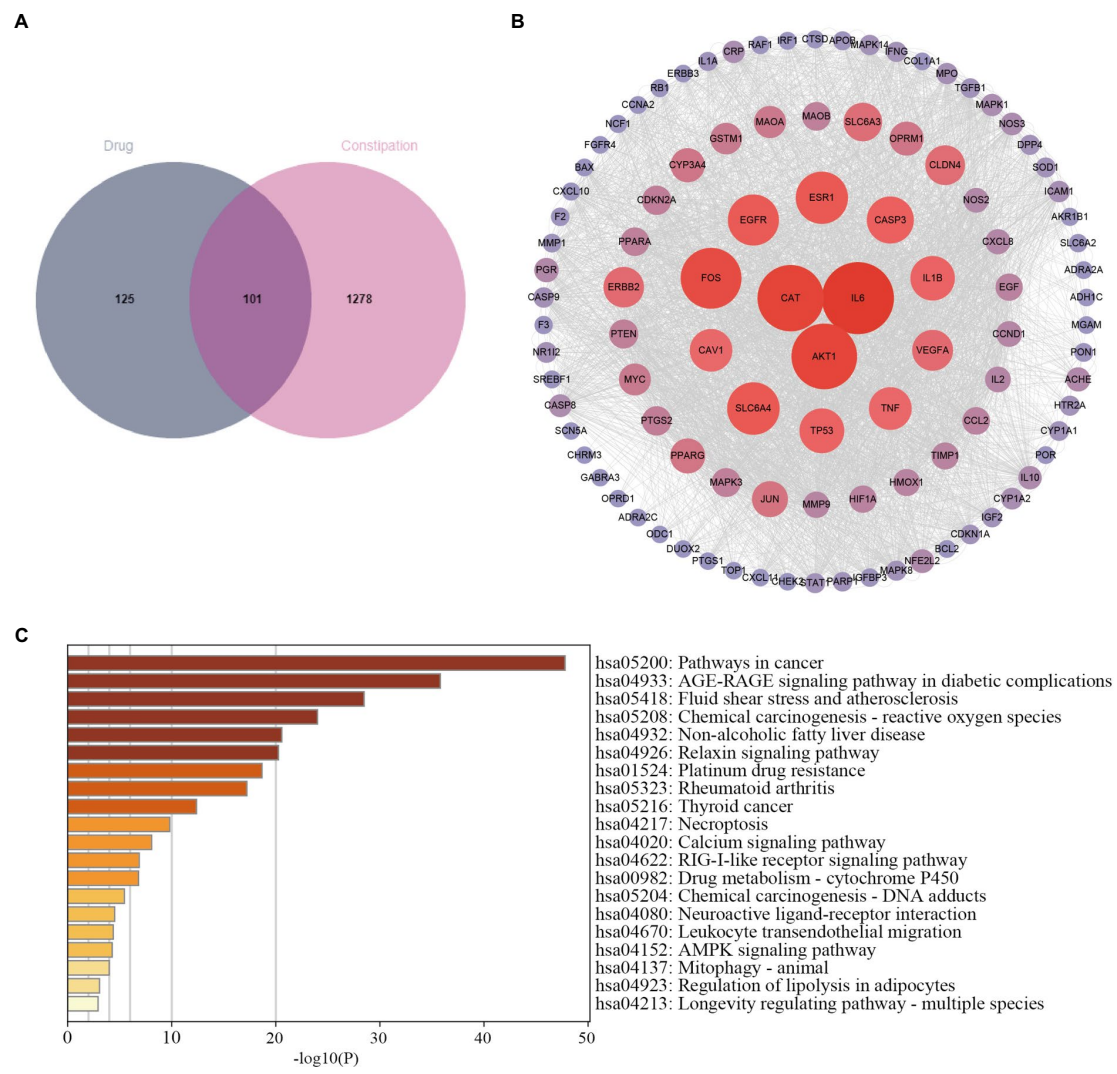
Pharmacological analysis of the network of SMD-constipation. **(A)** Venn diagram of the active ingredients of SMD and constipation. **(B)** Correlation network of SMD-constipation. **(C)** KEGG enrichment analysis.

We searched for 1,273 targets in the GeneCards database and 424 targets in the DisGeNET database. Totaling 1,379 targets after deduplication, Figure 1A shows 101 intersection targets between constipation and SMD.

#### 3.1.3. Protein–protein interaction (PPI) network analysis

The 101 target proteins were imported into the STRING database^5^ for analysis to obtain the PPI, and then Cytoscape 3. 7. 2 software was applied to visualize. The clustering coefficient in this PPI network was 0.7, the average number of neighboring nodes was 34.5, and there were 101 nodes and 1740 edges in the network (Figure 1B), which reflected good connectivity characteristics in the relationship network (Li et al., 2021d).

#### 3.1.4. Kyoto encyclopedia of genes and genomes pathway enrichment analysis results

The 101 signaling pathways were obtained from the KEGG pathway enrichment analysis, and the top 20 signaling pathways were visualized according to the number and significance of gene enrichment. Figure 1C shows that the signaling pathways involved in treating constipation with SMD are the cancer signaling pathway, AGE-RAGE signaling pathway in diabetic complications, reactive oxygen species, and non-alcoholic fatty liver disease. Therefore, we speculate that SMD may achieve the purpose of treatment through the pathways of cell information transmission, oxidative stress, inflammation, immune response, etc.

### 3.2. General characteristics

**Figure 2:**
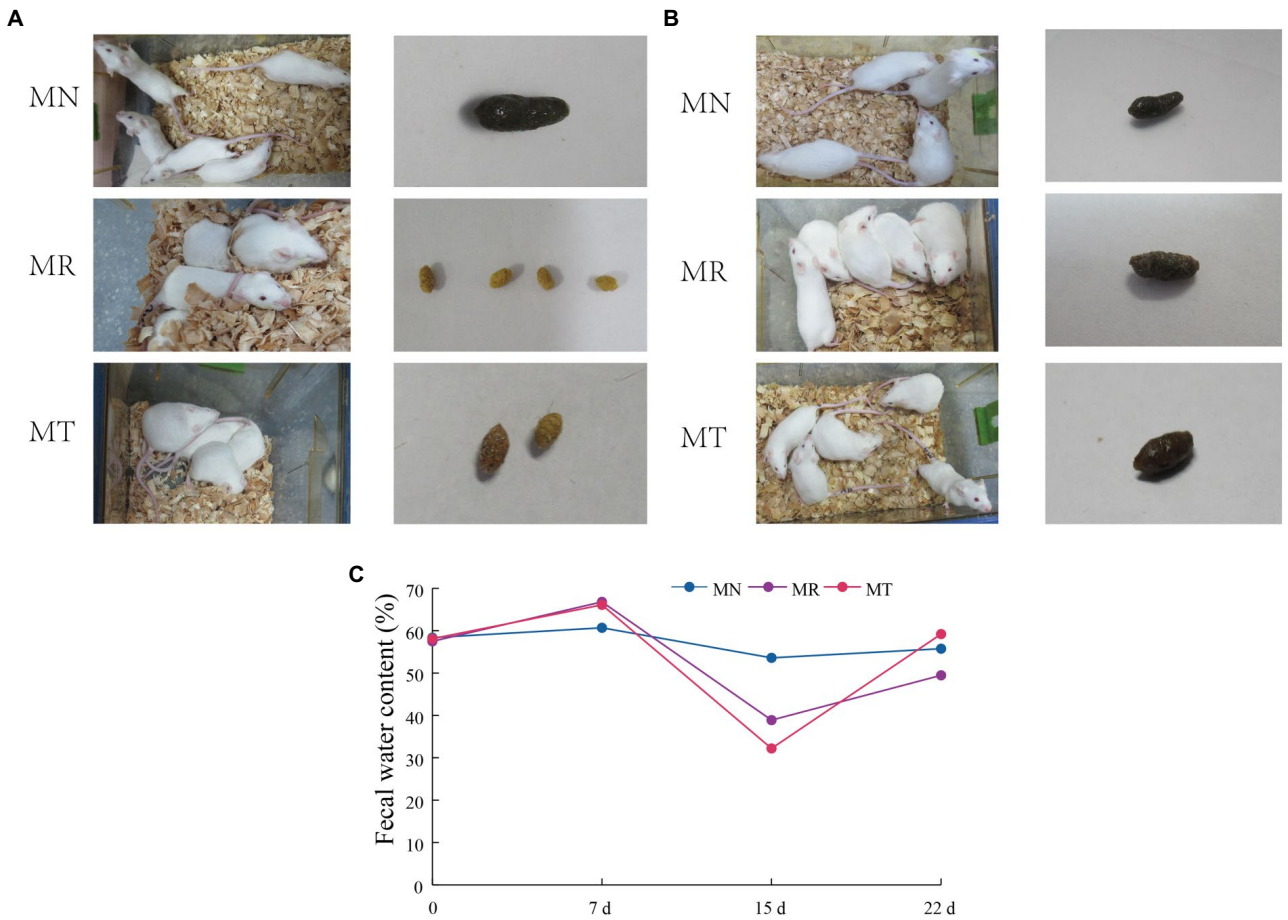
Behavioral changes, fecal characteristics and fecal water content in mice. **(A)** Molding stage. **(B)** SMD intervention Stage. **(C)** Fecal water content.

During the modeling of mice, the mice in MN group had smooth fur, bright eyes, and good autonomic activity. The mice in MR and MT groups had dull fur, squinting, and their autonomic activity was reduced, and liked to gather (Figure 2A).

When the mice were intervened with SMD, the mice in MN group still had smooth fur, bright eyes, and good mental state, the mice in MR group had vertical hair, dull eyes, poor mental state, and liked to gather, the mice in MT group had smooth fur, bright eyes and improved autonomic activity (Figure 2B).

### 3.3. Fecal characteristics of mice

Figures 2A,B shows that during molding, when gavaging *Folium sennae* decoction, the feces of mice in MR and MT group were shapeless, watery, perianal dirty, and with an obvious sour smell. However, on the morning of the 7th day of modeling, the feces of the model mice were formed before gavage, and the feces of the model mice were still not formed in the afternoon after gavage. When the diet and drinking water are controlled, the feces of mice in MR and MT groups are rough in appearance. When pressed by fingers, the feces are hard, with small particles, yellow, no sour smell, clean perianal and tail parts, and no humidity. During the administration stage, MR group did not get a good recovery, and the fecal characteristics of MT group were closer to those of MN group.

Figure 2C shows the fecal water content of the mice at each stage. Before modeling, the fecal water content of the three groups of mice was about 58%. After modeling, the fecal water content of mice in MR group (38.89%) and MT group (32.20%) was lower than that in MN group (53.59%). After SMD intervention, the fecal water content of mice in MN, MR and MT groups was 55.74, 49.46, and 59.20%, respectively. After SMD intervention, the fecal water content of mice in MN, MR and MT groups was 55.74, 49.46, and 59.20%, respectively. The fecal water content of mice in MT group is closer to that in MN group, which shows that after SMD intervention, the treatment of spleen deficiency constipation is better than that in MR group.

### 3.4. Weight changes of mice

**Figure 3:**
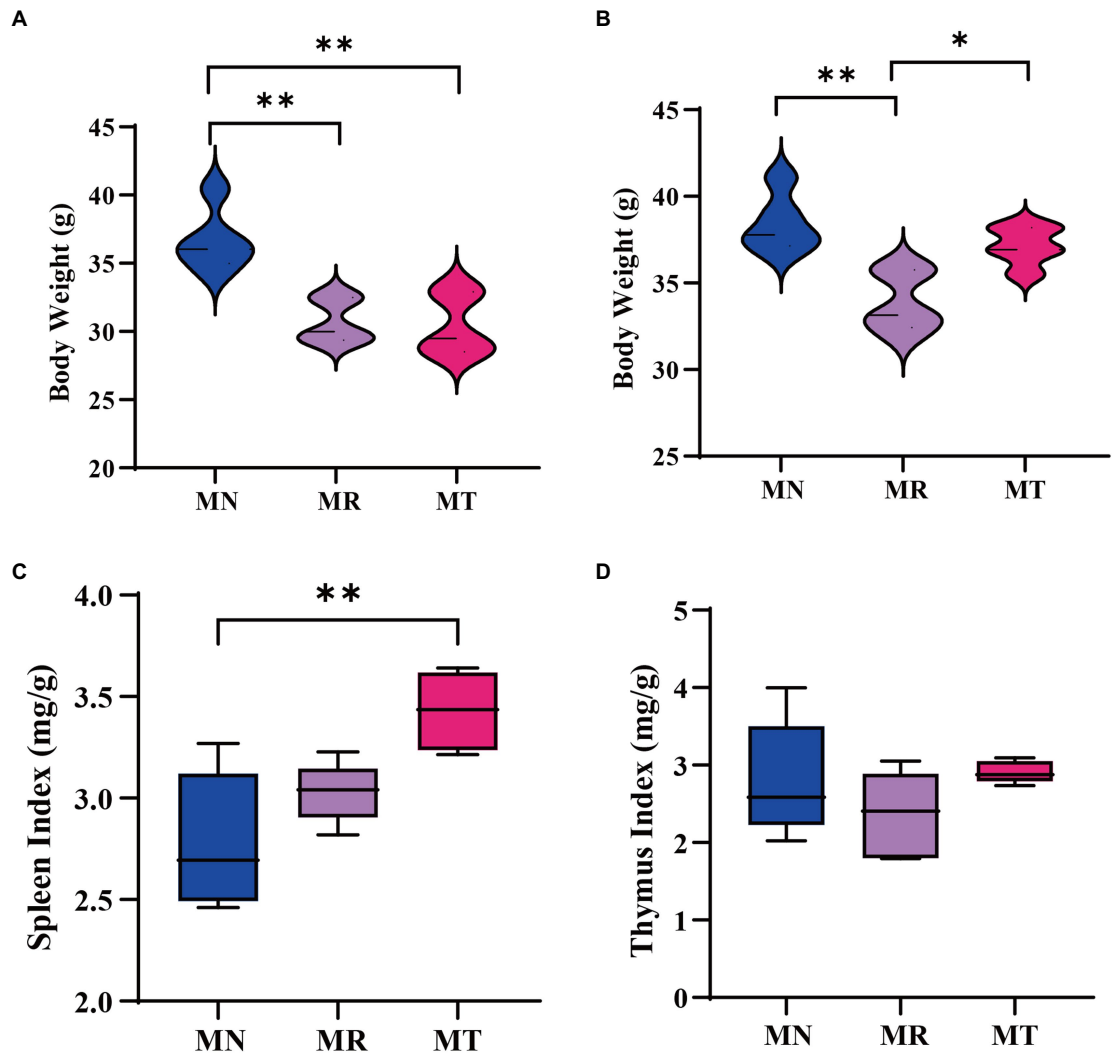
Effect of SMD on body weight and visceral index of mice with spleen deficiency constipation. **(A)** Change in body weight at the end of modeling. **(B)** Change in body weight at the end of drug administration. **(C)** Spleen index. **(D)** Thymus index.

Figure 3A shows the weight of mice in MR and MT groups was significantly lower than that in MN group at the modeling stage. After the administration, the weight of MR mice was significantly lower than that of MN and MT mice (Figure 3B), which indicated that SMD could recover the weight of mice.

### 3.5. Changes in the visceral index in mice

The visceral index is a preliminary index of immune function. The spleen and thymus are important immune organs in the body, and the spleen is the largest immune organ in the body, which plays an important role in defense and clearance (Li et al., 2020). The spleen and thymus index changes can directly reflect the changes in the body and cellular immunity (Li et al., 2022). It can be seen from Figures 3C,D that after the administration, the organ index of the MR group mice tends to decrease compared with those of MN and MT groups.

### 3.6. Serum VIP, 5-HT and liver MDA, SOD levels in mice

**Figure 4:**
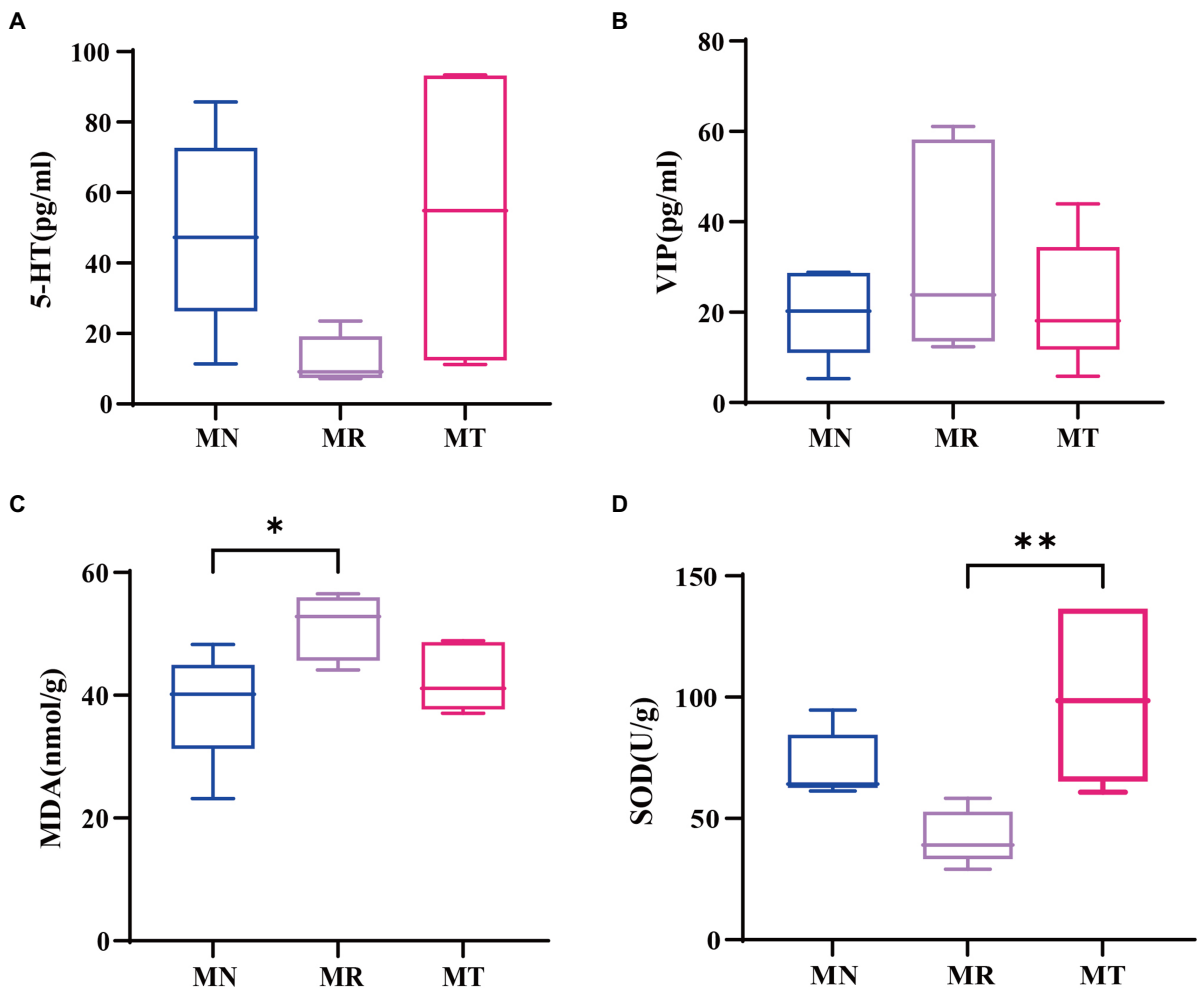
Effect of SMD on gastrointestinal hormones and oxidative stress indexes in mice with spleen deficiency constipation. **(A)** 5-HT content. **(B)** VIP content. **(C)** MDA content. **(D)** SOD content.

VIP and 5-HT belong to brain-gut peptide, and if 5-HT secretion is too little or VIP secretion is too much, can make the intestinal peristalsis slow, easy to cause constipation (Gershon, 2013; Iwasaki et al., 2019; Seo et al., 2019). Figures 4A,B shows the little difference between MN and MT, while a decreasing trend of 5-HT and an increasing trend of VIP in MR group. This indicated that intervention of SMD on the modeling of spleen deficiency constipation had better recovery of intestinal peristalsis function than natural recovery mice.

The abnormal metabolism of SOD and MDA *in vivo* has a strong cytotoxic effect on the body, including intestinal lining mucosal cells damage, intestinal mucosal dysfunction, and constipation (Xiang et al., 2019). The MDA content in MR group was significantly higher than that in MN group in Figure 4C, while the difference in MDA content between MT and MN groups was not significant. In Figure 4D, SOD content of the MN group was significantly lower than that of MT group, and SOD content of the MT group was not significantly different from that of MN group. The above indicates that MR group produced more lipid oxidation end products. It suggests that the mice with spleen deficiency and constipation suffered from oxidative stress damage *in vivo*. While SMD may enhance the antioxidant effect and reduce of lipid oxidation end products.

### 3.7. Effect of SMD on fecal microbial activity in mice with spleen deficiency constipation

**Figure 5:**
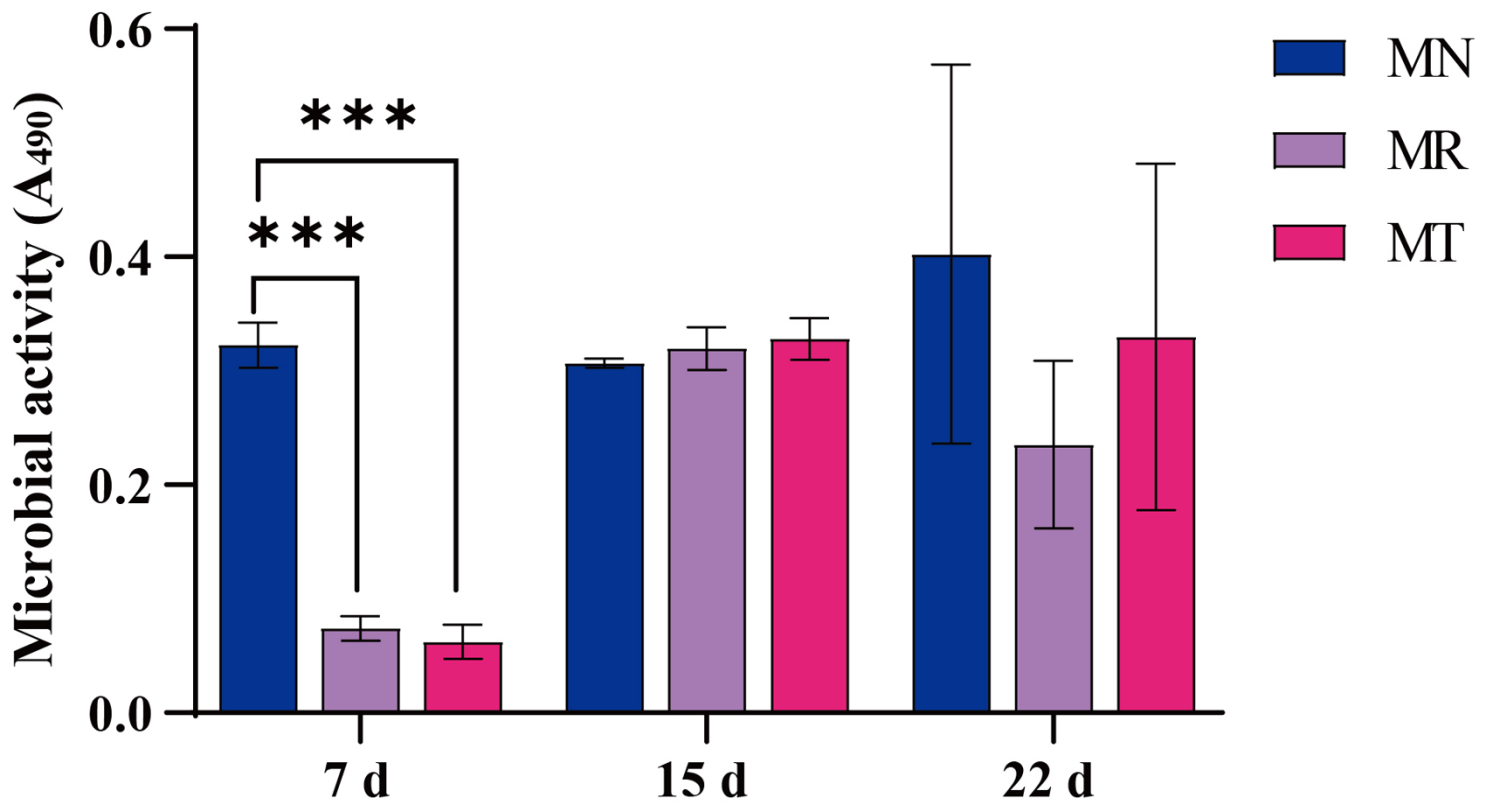
Effect of SMD on fecal microbial activity in mice with spleen deficiency constipation.

FDA can be catalyzed by nonspecific enzymes expressed in bacteria and fungi to hydrolyze fluorescein. The metabolic capacity of microorganisms can be reflected by measuring intestinal microbial activity *in vitro* (Bokulich et al., 2013). Figure 5 shows the microbial activity of feces under the condition of absorbance of 490 nm. Seventh day is the last day of *Folium sennae* decoction gavage, and the microbial activity of MR and MT groups is significantly lower than that of MN group. Fifteenth day is the last day of modeling, and the microbial activity of MR and MT groups tends to increase compared with MN group. Twenty-second day is the last day of the gavage of SMD, the microbial activity of MR group tends to decrease compared with MN and MT groups.

### 3.8. Analysis of the number and diversity of intestinal mucosal bacteria OTUs in mice

**Figure 6:**
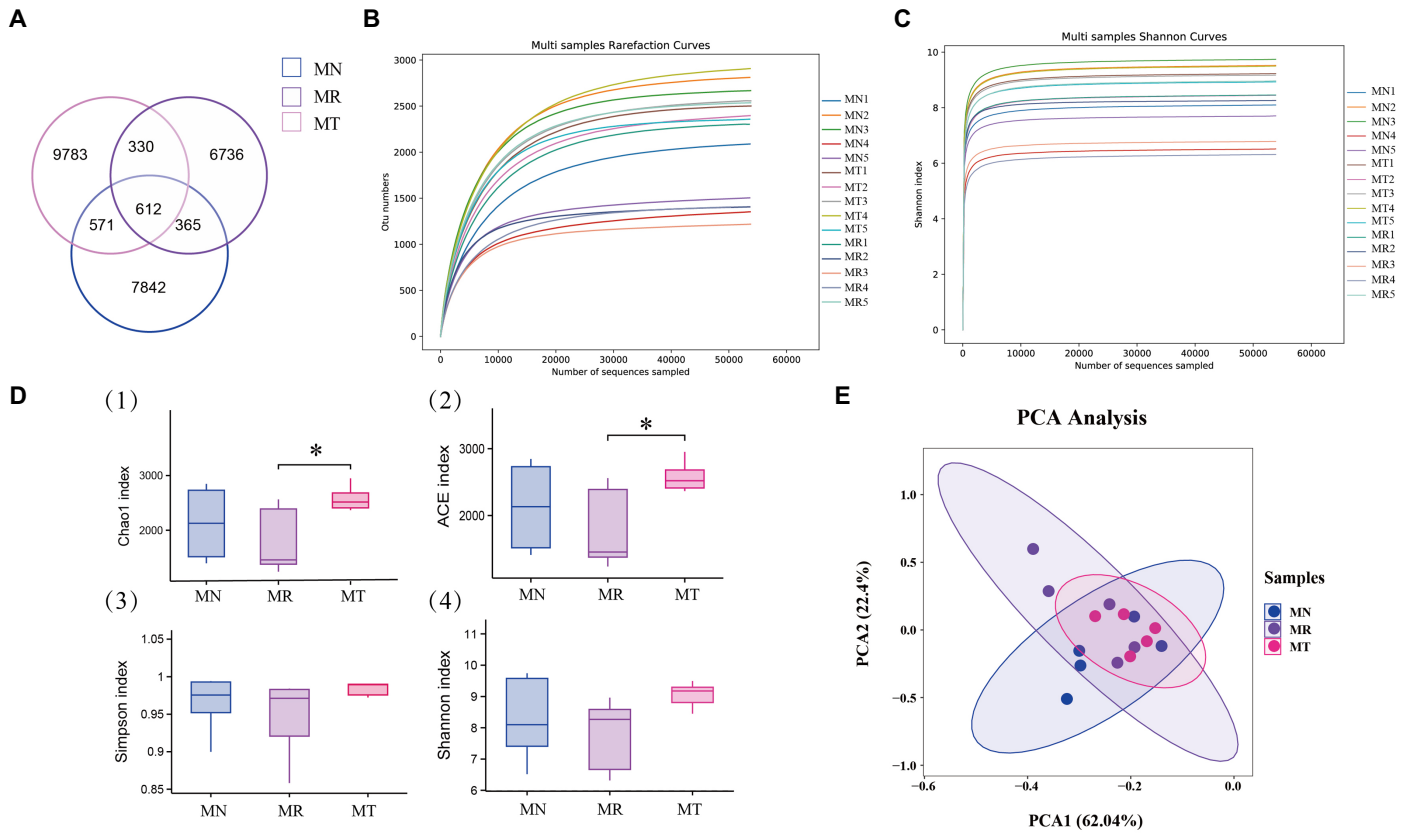
Effect of SMD on the number and diversity of bacterial OTUs in the intestinal mucosa of mice with spleen deficiency constipation. **(A)** Venn diagram: number distribution of OTUs of three groups of intestinal mucosal bacteria. **(B)** Dilution curve **(C)** Shannon-Wiener curve. **(D)** Alpha diversity index (Sharma and Rao, 2017) Chao1. (Milosavljevic et al., 2022) ACE. (Zhao et al., 2022) Simpson. (Tian et al., 2021) Shannon. **(E)** Principal component analysis.

The total OTU numbers of intestinal mucosa of mice in MN, MR, and MT groups were 9,390, 8,043, and 11,296, respectively. The intersection OTU number of the three groups was 612 (Figure 6A), which indicated that the natural recovery after spleen deficiency constipation in mice the number of mucosal bacterial OTU was reduced. The number of intestinal mucosal bacterial OTU in mice increased significantly after SMD intervention. There were individual differences among all groups in the dilution curves. The dilution curves leveled off in all three groups. It indicated that the amount of sequencing data was sufficient to estimate information on the majority of microbial species in the samples (Figure 6B). In combination with the Alpha diversity analysis, the Shannon-Wiener curves all flattened out, it also indicated that the amount of sequencing data was large enough to mirror the information of most microbial species in the samples (Figure 6C). In the Alpha diversity index (Figure 6D), the Chao1 and ACE indices were significantly higher in MT group than in MR group, while the differences between MT and MN groups were insignificant. Compared with MN and MT groups, the Shannon and Simpson indices in MR group tended to decrease, but the differences between the three groups were not significant. These results suggest that MR group decreased intestinal mucosal bacterial diversity. At the same time, intestinal mucosal bacterial diversity increased after spleen deficiency constipation modeling with the intervention of SMD. Beta diversity showed 62.04% for principal coordinate variable 1 and 22.4% for principal coordinate variable 2 (Figure 6E). From the results, the samples of MN and MT groups were evenly distributed and relatively concentrated. This means they have higher similarity in bacterial community composition. Compared with other groups, the distribution of samples in MR group was relatively scattered, indicating some differences in the structure of the bacterial community.

### 3.9. Analysis of the relative abundance of intestinal mucosal bacteria in mice

**Figure 7:**
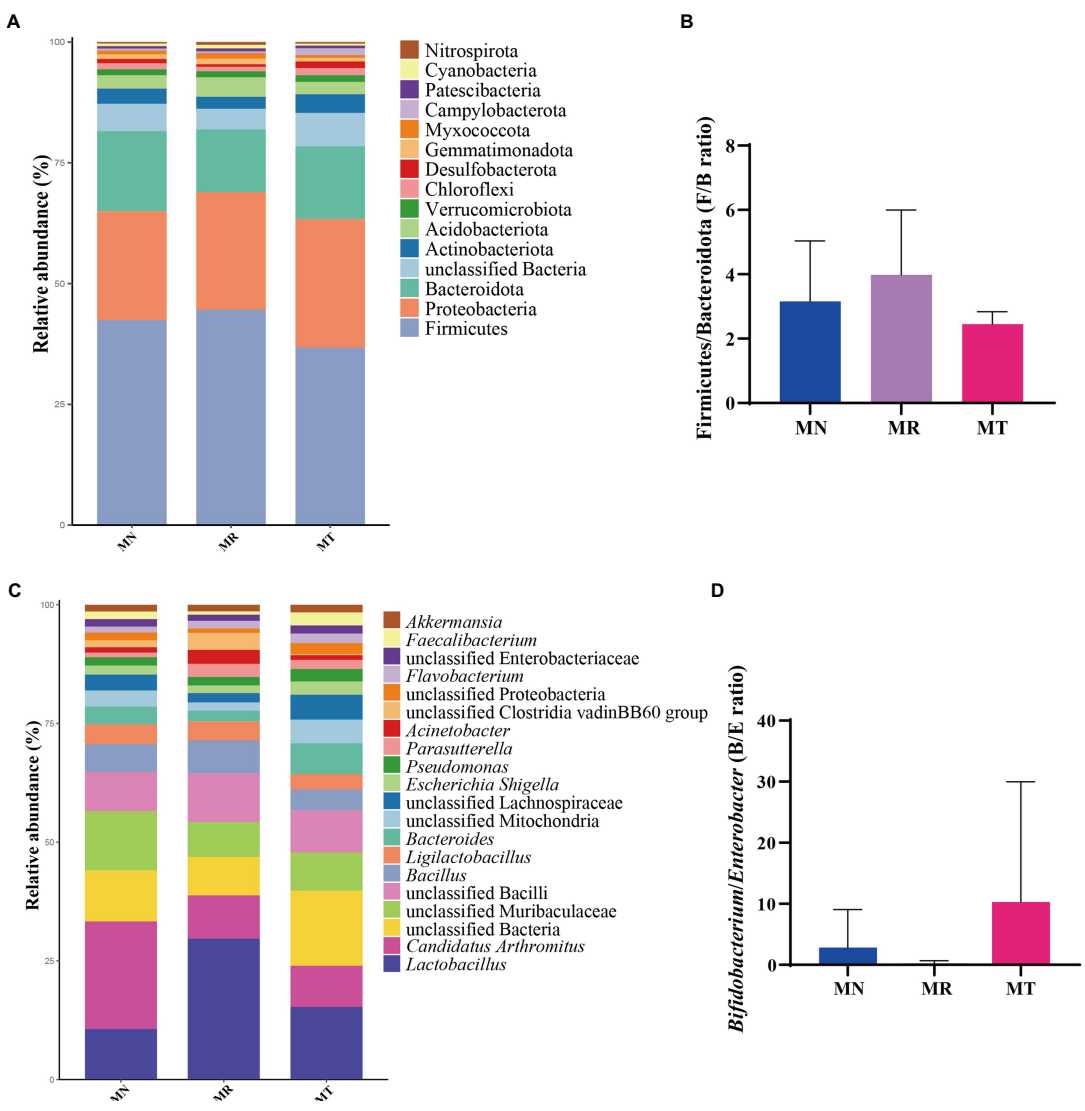
Effect of SMD on the relative abundance of bacteria in the intestinal mucosa of mice with spleen deficiency constipation. **(A)** Relative abundance at the phylum level. **(B)** Histogram of the Firmicutes/Bacteroidota (F/B) ratio. **(C)** Relative abundance at the genus level. **(D)** *Histogram of the Bifidobacterium/Enterobacter (B/E) ratio.*

Figure 7A shows that Firmicutes was the dominant phylum in all three groups, followed by Proteobacteria, Bacteroidota, and unclassified Bacteria. There was an increasing trend in the relative abundance of Firmicutes (44.59%) in MR group compared to MN group (42.42%), but in MT group (36.77%) had a decreasing trend, indicating that natural recovery after molding stimulates the growth of Firmicutes and that SMD has a regulatory effect on Firmicutes. The relative abundance of Proteobacteria showed an increasing trend in MR (24.36%) and MT (26.57%) groups compared to MN group (22.54%). There was a decreasing trend of Bacteroidota in the MR group (12.96%) compared to MN (16.58%) and MT (15.05%) groups, suggesting that SMD regulates the abundance of Bacteroidota to normal levels by promoting its growth. Figure 7B shows the histogram of the Firmicutes/Bacteroidota (F/B) ratio, MR group showed an increasing trend of F/B value, but MT group showed a decreasing trend.

Figure 7C demonstrates the relative abundance of mouse mucosal bacteria at the genus level, with the top five dominant bacteria in each of the three groups being *Lactobacillus*, *Candidatus Arthromitus*, unclassified Bacteria, unclassified *Muribaculaceae*, and unclassified Bacilli. There was a tendency for *Lactobacillus* to rise in the intestinal mucosa in MR group (29.70%) and MT group (15.29%) compared to MN group (10.68%), with MT group tending more toward MN group, suggesting that the tetracycline intervention contributed to the balance of the intestinal microbiota. The relative abundance of *Candidatus* Arthromitus in MR (9.08%) and MT (8.68%) groups tended to decrease compared to MN group (22.62%). It suggests that modeling may inhibit the growth of *Candidatus* Arthromitus and that Tetragrammaton restores it more slowly. Figure 7D shows the histogram of the *Bifidobacterium/Enterobacter* (B/E) ratio. There is a decreasing trend in MR group and an increasing trend in MT group.

### 3.10. Analysis of characteristic bacteria of the intestinal mucosa in mice

**Figure 8:**
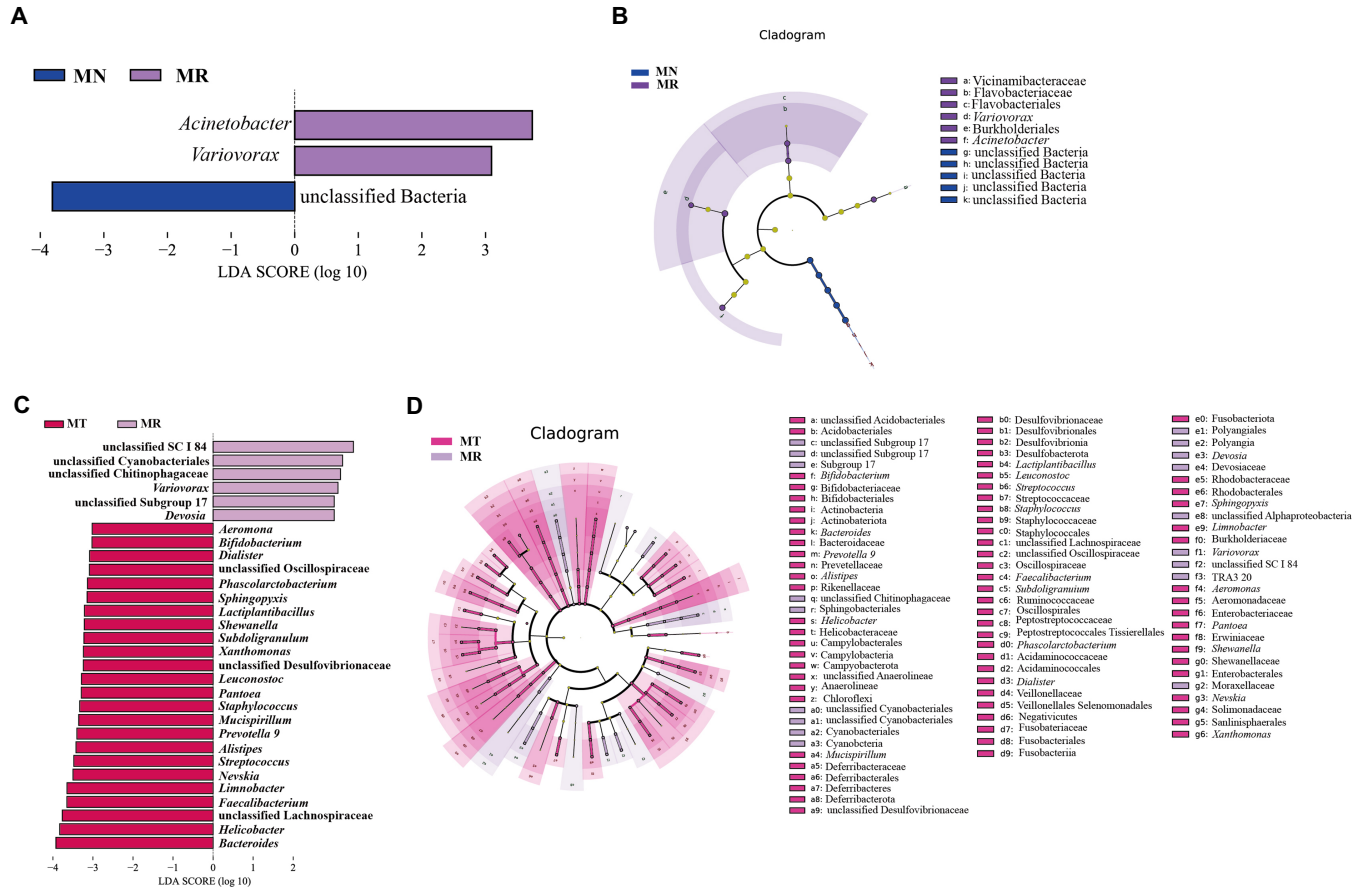
Effect of SMD on characteristic bacteria of the intestinal mucosa in mice with spleen deficiency constipation. **(A)** LDA score plots for MN and MR groups. **(B)** LEfSe analysis of MN and MR groups. **(C)** LDA score plots of MR and MT groups. **(D)** LEfSe analysis of MR and MT groups.

The LDA and LEfSe analyzes at a threshold of 3 were chosen. Figures 8A,B compares the characteristic bacteria in MN and MR groups, with unclassified Bacteria in MN and two characteristic bacteria containing *Acinetobacter* and *Variovorax* in MR group. Figures 8C,D compares the characteristic bacteria in MR and MT groups, with 6 in MR group and 24 in MT group. Among them, MR group had an increased abundance of *Devosia, Variovorax,* and some unclassified genera, and MT group was characterized by an increased abundance of *Bacteroides*, *Helicobacter, Faecalibacterium*, *Limnobacter*, *Nevskia*, *Alistipes*, *Subdoligranulum*, *Lactiplantibacillus*, *Phascolarctobacterium*, and other genera. The results showed a significant difference in the intestinal mucosal microbiota between MR and MT groups. The above results indicated significant differences in the differential bacteria among the three groups in different classification systems.

### 3.11. Correlation analysis

**Figure 9:**
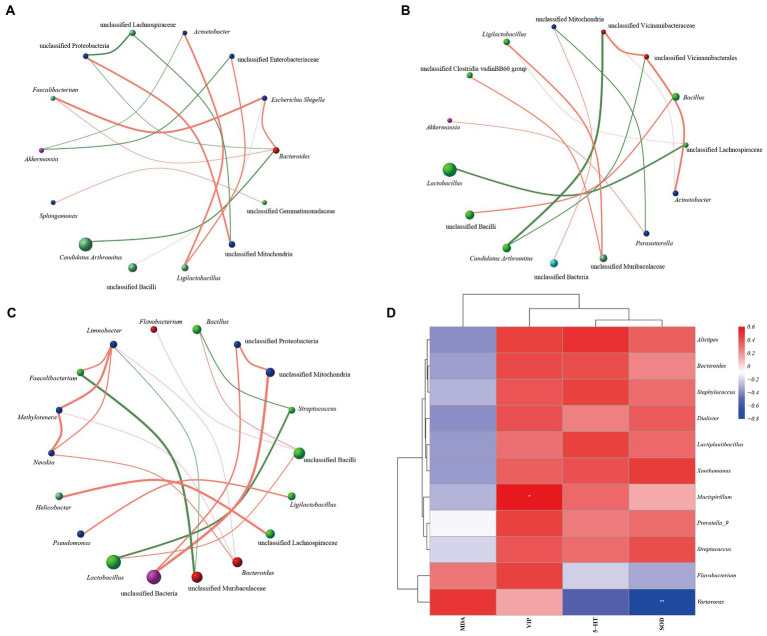
Correlation network analysis of intestinal mucosal microbiota in mice with spleen deficiency constipation intervened by SMD. **(A)** Interaction network between the microbiota of MN group. **(B)** Interaction network between the microbiota of MR group. **(C)** Interaction network between the microbiota of MT group. **(D)** Heat map of correlation analysis between microbiota and indicators.

Combined with the correlation coefficient analysis, we constructed the interaction networks of “microbiota-microbiota” and “microbiota-indicator” among the groups, respectively. Figures 9A–C shows the interaction network between the groups of microbiota. *Candidatus Arthromitus* is negatively correlated with Bacteroides in MN group, and *Faecalibacterium* is positively correlated with *Escherichia Shigella*. *Arthromitus* was negatively correlated with unclassified Vicinamibacteraceae, *Faecalibacterium* was negatively correlated with unclassified Muribaculaceae in MT group, and *Methylotenera* was negatively correlated with *Nevskia* and *Limnobacter* were positively correlated. It showed that MN, MR, and MT groups underwent different interactions in different microbial ecological niches. Figure 9D shows that *Variovorax* was significantly negatively correlated with SOD and *Mucispirillum* was significantly positively correlated with VIP. It is suggested that the interaction between the above factors may be involved in regulating intestinal mucosal microbiota in mice with spleen deficiency constipation by SMD.

### 3.12. Kyoto encyclopedia of genes and genomes enrichment analysis

**Figure 10:**
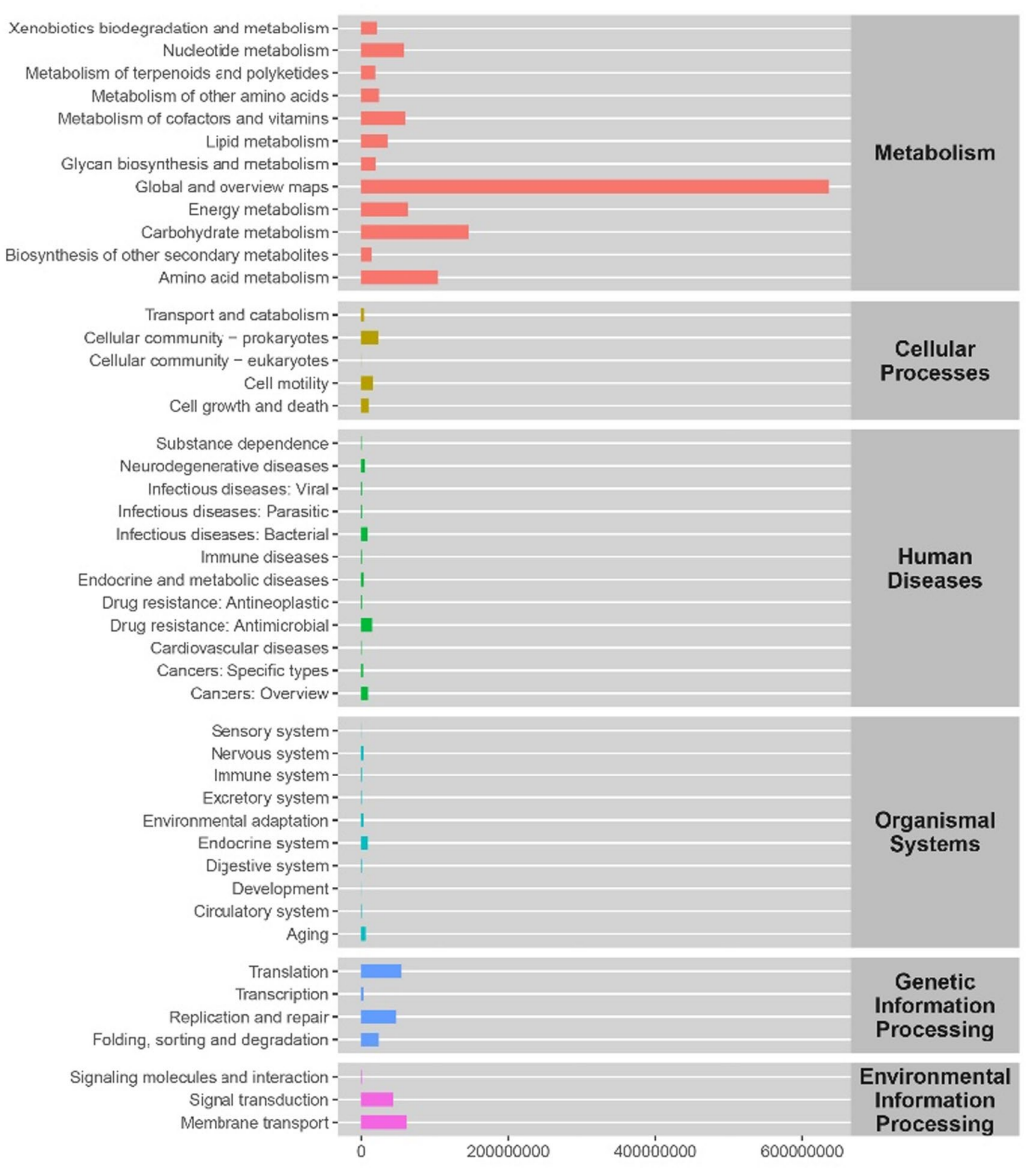
The predicted functional abundance of intestinal mucosal microbiota KEGG in mice with spleen deficiency constipation during SMD intervention.

The change of intestinal flora relationship also indicates the change of the function. Therefore, we made a functional prediction. We analyzed six KEGG metabolic pathways, including metabolism, genetic information processing, environmental information processing, cell processes, biological systems and human diseases. Figure 10 counts the abundance of secondary functional pathways in the current KEGG database, and the second category contains a total of 46 metabolic pathway sub-functions. The pathways with higher abundance in metabolism are global and overview maps, carbohydrate, and amino acid metabolism. Those with higher abundance in genetic information processing are Translation, Replication, and repair, and those with higher abundance in environmental information processing are Signal transduction, Membrane transport. We speculate that these metabolic pathways may be the main pathways of the change of intestinal mucosal microbiota in mice with spleen deficiency constipation treated with SMD.

## 4. Discussion

Intestine is the largest digestive organ in the body and one of the largest organs in contact with the outside world. The number of intestinal mucosal microbiota was accompanied by pathological changes such as disruption of intestinal mucosal tissue, reducing expression of tight junction proteins, and increasing intestinal mucosal permeability, which can affect the barrier function of the intestinal mucosa (Zhou et al., 2020). TCM has a long history by focusing on the overall adjustment of the body and can promote intestinal microecological balance by increasing the diversity of intestinal microbiota (Li et al., 2021). SMD is widely used in China to treat gastrointestinal diseases such as abdominal distension, abdominal pain, diarrhea, and constipation. It is found that the adjuvant treatment of SMD for constipation in children can better improve the imbalance of intestinal flora in children, promote the reproduction of probiotics, and be more conducive to regulating the balance of intestinal flora (Fang, 2019).

In our animal experiment, spleen deficiency constipation could cause weight loss, while the weight increased significantly after SMD intervention, indicating that SMD could help weight recovery, this is probably because SMD has the function of invigorating spleen and stomach, increasing the appetite, and improving its digestion and absorption functions (Liu, 2014). The thymus and spleen are important for establishing the body’s immune function (Li et al., 2022c). Therefore, after the intervention of SMD, we examined the spleen and thymus indices. The spleen and thymus indices of the natural recovery mice were low after modeling. The spleen and thymus indices were elevated after the SMD intervention, indicating that SMD can increase immunity.

Our biochemical index analysis showed a decreasing trend of serum VIP and an increasing trend of 5-HT in MR group, it indicates that the individual intestinal motility of the natural recovery mice after spleen deficiency constipation might not be fully recovered and the motility of peristalsis was still slow. The MDA content of mice in MR group was higher than MN group. Meanwhile, the SOD content in MR group was lower than MT group. This suggests that the mice with spleen deficiency constipation were damaged by oxidative stress, SMD could act as an antioxidant, allowing the mice with spleen deficiency constipation to recover from oxidative stress damage, which is similar to the antioxidant capacity predicted by the pharmacological function of our network.

FDA hydrolase activity can reflect the metabolic capacity of intestinal microorganisms (Huang et al., 2022). In our results, the microbial activity of the modeled mice was significantly decreased in all the stages of *Folium sennae* decoction gavage, which may be due to the antibacterial effect of *Folium sennae* (Sun and Zhang, 2017). The microbial activity of the modeled mice increased during the constipation phase, probably because constipation caused a long-term accumulation of feces in the intestine, increasing pathogenic bacteria’s growth (Zhang et al., 2021). The microbial activity of mice in MR group decreased during the administration phase. We speculate that natural recovery may have inhibited the growth of certain microorganisms or the expression of their functional genes, resulting in an imbalance in the intestinal microbiota. The microbial activity of mice in MT group was higher than that in MR group, but lower than that in MN group, which we thought that SMD might promote the growth of certain microbiota or the expression of its functional genes to a certain extent. It requires further analysis in conjunction with later intestinal mucosal bacteria changes in mice.

There were more OTUs unique to the intestinal mucosa of mice in MT group than in MR group, but lower than MN group, suggesting changes in the intestinal mucosa bacteria of mice after natural recovery and SMD intervention. We investigated the effects of SMD on the diversity, community structure, and function of the intestinal mucosal microbiota in mice with spleen deficiency constipation. Compared with MN group, all the Alpha diversity indices in MR group showed a decreasing trend, indicating that the intestinal mucosal diversity in MR group of mice was decreased. Moreover, the ACE and Chao1 indices in MR group were significantly lower than those in MT group, and the Shannon and Simpson indices showed a decreasing trend. It indicates that the intestinal mucosal diversity increased in mice with spleen deficiency constipation after intervention with SMD. PCA analysis indicated differences in the community structure of intestinal mucosal microbiota in different groups of mice. Thus, we hypothesize that SMD affects the diversity and changes of microbial communities in mice with spleen deficiency constipation. Further insight into the effect of SMD on the intestinal microbial environment was obtained by comparing the relative abundance of the group to group.

The F/B value is often used to measure intestinal homeostasis, and the increase of the F/B value usually indicates the imbalance of intestinal microbiota (Shin et al., 2015). The B/E value indicates intestinal colonization resistance, and the decrease of the B/E value means the risk of intestinal pathogenic bacteria invasion is greatly increased (Shin et al., 2015). In the experimental results, the F/B values of MR group were higher than MT and MN groups, and the tendency for the B/E values to decrease in MR group and to increase in MT group, it was suggested that when the spleen deficiency constipation natural recovery, the intestinal mucosa of mice were more vulnerable to the attack of pathogenic bacteria, while SMD may had the effect of enhancing immunity and regulating the balance of intestinal microbiota, improving the ability of mice intestine to resist the attack of pathogenic bacteria (Li et al., 2020). At the same time, it was confirmed by the increased abundance of beneficial bacteria such as *Bacteroides*, *Faecalibacterium*, *Alistipes*, *Subdoligranulum*, *Lactiplantibacillus*, and *Phascolarctobacterium* in MT group. Among them, *Bacteroides* have a robust polysaccharide degradation system, the polysaccharide utilization system of *Bacteroides* enhances its ecological adaptability by securing a broad range of target polysaccharides, especially undigested dietary fiber (Tan et al., 2019). *Faecalibacterium* is a genus of bacteria that produces short-chain fatty acids that benefit humans (Nishiwaki et al., 2020; Qian et al., 2020). *Alistipes* are anaerobic bacteria found mainly in the gastrointestinal microbiota of healthy humans and may have protective effects against diseases such as liver fibrosis, colitis, cancer immunotherapy and cardiovascular diseases (Parker et al., 2020). *Subdoligranulum* can benefit necrotizing small intestinal colitis by affecting butyrate production (Lin et al., 2021). *Lactiplantibacillus* is a probiotic known to increase the diversity and function of intestinal microbiota and reduce Enterobacteriaceae’s survival (Linninge et al., 2019). *Phascolarctobacterium* can also act as a genus of beneficial bacteria to regulate intestinal health (Yuan et al., 2020). We speculate from these results that SMD may regulate intestinal health and relieve constipation by increasing the growth of beneficial bacteria.

In addition, the correlation analysis showed that *Variovorax* was significantly negatively correlated with SOD and *Mucispirillum* was significantly positively correlated with VIP. We speculate that *Variovorax* inhibits the secretion of SOD, which decreases the body’s antioxidant capacity. *Mucispirillum* enhances the secretion of VIP, which may decrease the gastrointestinal tract’s peristalsis. The interrelationship between the various bacterial microbiota may also be one of the mechanisms by which SMD works. The highest abundance of global and overview maps of metabolic pathways in functional prediction may be a new direction for further research on treating spleen deficiency constipation with SMD in the future.

Therefore, based on the content of 5-HT and VIP, intestinal mucosal microbiota, and the correlation analysis of brain-gut peptides and intestinal microbiota, we speculated that there was a certain relationship between the intestinal flora and the brain-gut axis. Thus, SMD may relieve constipation through the brain-bacteria-gut axis.

## 5. Conclusion

In summary, the diversity and abundance of intestinal microbiota and microbial interactions appear to be key factors in the regulation of intestinal health. We speculate that the mechanism of SMD relieving constipation increases the growth of beneficial bacteria and is associated with brain-bacteria-gut axis. At the same time, the regulation of SMD on oxidative stress level is also one of the functions of treating constipation, which provides a basis for the future regulation of intestinal microecology by SMD.

## Data availability statement

The datasets presented in this study can be found in online repositories. The names of the repository/repositories and accession number(s) can be found at: https://www.ncbi.nlm.nih.gov/, PRJNA879101.

## Ethics statement

Animal experiments were conducted under animal protocols approved by the Animal Ethics and Welfare Committee of the Hunan University of Chinese Medicine (protocol number: LL2022052505). All animal work was carried out following the guidelines of the Institutional Animal Care and Use Committee of the Hunan University of Chinese Medicine. This study was carried out in compliance with the ARRIVE guidelines.

## Author contributions

XY: data analysis and writing the original draft. ND and YC: review and editing. KZ and XY: performing animal experiments. XP and ZT: project administration, review, and funding acquisition. All authors contributed to the article and approved the submitted version.

## Funding

This research was financially supported by the Natural Science Foundation of Hunan Province (No. 2022JJ40332) and the Domestic First-class Discipline Construction Project of Chinese Medicine of Hunan University of Chinese Medicine (4901–020000200207).

## Conflict of interest

The authors declare that the research was conducted in the absence of any commercial or financial relationships that could be construed as a potential conflict of interest.

## Publisher’s note

All claims expressed in this article are solely those of the authors and do not necessarily represent those of their affiliated organizations, or those of the publisher, the editors and the reviewers. Any product that may be evaluated in this article, or claim that may be made by its manufacturer, is not guaranteed or endorsed by the publisher.

## Footnotes

^1^ https://www.disgenet.org/

^2^ https://www.genecards.org/

^3^ https://qiime2.org/

^4^ http://tcmspw.com/index.php

^5^ https://cn.string-db.org/

## Abbreviations

SMD: Simo decoction
CL: *Citrus aurantium* L
AD: *Aucklandia lappa* Decne
AL: *Areca catechu* L
LK: *Lindera aggregata* (Sims)Kos-term
TCM: Traditional Chinese medicine
5-HT: 5-Hydroxytryptamine
VIP: Vasoactive Intestinal Peptide
MDA: Malondialdehyde
SOD: Superoxide Dismutase
FDA: Fluorescein diacetate
PPI: Protein–Protein Interaction
OTU: Operational taxonomic units
PCA: Principal Component Analysis
F/B: *Firmicutes*/Bacteroidota
B/E: *Bifidobacterium*/*Enterobacter*
LDA: Linear Discriminant Analysis
LEfSe: Linear discriminant analysis Effect Size
KEGG: Kyoto Encyclopedia of Genes and Genomes
ELISA: Enzyme-linked immunosorbent assay
